# Meningococcal carriage 7 years after introduction of a serogroup A meningococcal conjugate vaccine in Burkina Faso: results from four cross-sectional carriage surveys

**DOI:** 10.1016/S1473-3099(20)30239-5

**Published:** 2020-12

**Authors:** Sarah Mbaeyi, Emmanuel Sampo, Kambiré Dinanibè, Issaka Yaméogo, Malika Congo-Ouédraogo, Mamadou Tamboura, Guetawendé Sawadogo, Kalifa Ouattara, Mahamadou Sanou, Tanga Kiemtoré, Gerard Dioma, Barnabé Sanon, Hermann Somlaré, Augustin Kyetega, Absatou Ky Ba, Flavien Aké, Félix Tarbangdo, Frederic Acho Aboua, Yvette Donnou, Idrissa Kamaté, Jaymin C Patel, Susanna Schmink, Michael W Spiller, Nadav Topaz, Ryan Novak, Xin Wang, Brice Bicaba, Lassana Sangaré, Rasmata Ouédraogo-Traoré, Paul A Kristiansen

**Affiliations:** a National Center for Immunization and Respiratory Diseases, US Centers for Disease Control and Prevention, Atlanta, GA, USA; b Hôpital Schiphra Protestant, Ouagadougou, Burkina Faso; c Centre Hospitalier Universitaire Pédiatrique Charles de Gaulle, Ouagadougou, Burkina Faso; d Direction de la Protection de la Santé de la Population, Burkina Faso Ministry of Health, Ouagadougou, Burkina Faso; e Centre Hospitalier Universitaire de Yalgado Ouédraogo, Ouagadougou, Burkina Faso; f Centre Hospitalier Régional de Kaya, Kaya, Burkina Faso; g Centre Hospitalier Universitaire du Bogodogo, Ouagadougou, Burkina Faso; h Davycas International, Gounghin Petit-Paris, Ouagadougou, Burkina Faso; i World Health Organization, Intercountry Support Team, Ouagadougou, Burkina Faso; j Norwegian Institute of Public Health, Skøyen, Oslo, Norway

## Abstract

**Background:**

In the first 2 years after a nationwide mass vaccination campaign of 1–29-year-olds with a meningococcal serogroup A conjugate vaccine (MenAfriVac) in Burkina Faso, carriage and disease due to serogroup A *Neisseria meningitidis* were nearly eliminated. We aimed to assess the long-term effect of MenAfriVac vaccination on meningococcal carriage and herd immunity.

**Methods:**

We did four cross-sectional studies of meningococcal carriage in people aged 9 months to 36 years in two districts of Burkina Faso between May 2, 2016, and Nov 6, 2017. Demographic information and oropharyngeal swabs were collected. Meningococcal isolates were characterised using whole-genome sequencing.

**Findings:**

Of 14 295 eligible people, 13 758 consented and had specimens collected and laboratory results available, 1035 of whom were meningococcal carriers. Accounting for the complex survey design, prevalence of meningococcal carriage was 7·60% (95% CI 5·67–9·52), including 6·98% (4·86–9·11) non-groupable, 0·48% (0·01–0·95) serogroup W, 0·10% (0·01–0·18) serogroup C, 0·03% (0·00–0·80) serogroup E, and 0% serogroup A. Prevalence ranged from 5·44% (95% CI 4·18–6·69) to 9·14% (6·01–12·27) by district, from 4·67% (2·71–6·64) to 11·17% (6·75–15·59) by round, and from 3·39% (0·00–8·30) to 10·43% (8·08–12·79) by age group. By clonal complex, 822 (88%) of 934 non-groupable isolates were CC192, all 83 (100%) serogroup W isolates were CC11, and nine (69%) of 13 serogroup C isolates were CC10217.

**Interpretation:**

Our results show the continued effect of MenAfriVac on serogroup A meningococcal carriage, for at least 7 years, among vaccinated and unvaccinated cohorts. Carriage prevalence of epidemic-prone serogroup C CC10217 and serogroup W CC11 was low. Continued monitoring of *N meningitidis* carriage will be crucial to further assess the effect of MenAfriVac and inform the vaccination strategy for future multivalent meningococcal vaccines.

**Funding:**

Bill & Melinda Gates Foundation and Gavi, the Vaccine Alliance.

## Background

In the meningitis belt of sub-Saharan Africa, *Neisseria meningitidis* serogroup A has historically caused high rates of endemic disease and large-scale epidemics.^1^ In 2010, Burkina Faso became the first country to introduce a serogroup A meningococcal conjugate vaccine (MenAfriVac) through mass vaccination of 1–29-year-olds, achieving 96% coverage.^2^ In the 2 years after the mass vaccination campaign, carriage and disease due to serogroup A were nearly eliminated in Burkina Faso, showing the impact of the vaccine on carriage, disease, and herd immunity.^3^, ^4^ However, in 2014–15, five cases of serogroup A meningococcal meningitis, as well as several cases of serogroup C meningococcal meningitis caused by a strain with molecular characteristics similar to the sequence type responsible for large meningococcal meningitis outbreaks in Niger and Nigeria (ST-10217), were reported in Burkina Faso; most of the serogroup A cases, along with several serogroup C cases, were detected in Ouahigouya district.^5^, ^6^, ^7^, ^8^, ^9^

Despite a more than 99% reduction in the incidence of serogroup A disease in MenAfriVac-vaccinated countries,^10^ concerns about possible recrudescence of serogroup A *N meningitidis* in Burkina Faso resulting from waning MenAfriVac-induced immunity and susceptibility of unvaccinated new-birth cohorts, as well as emergence of novel, epidemic-prone strains of non-serogroup A *N meningitidis*, motivated implementation of a follow-up meningococcal carriage study during 2016–17.

In this report, we describe the prevalence and characteristics of meningococcal carriage in Burkina Faso 7 years after the mass MenAfriVac vaccination campaign and during the period of MenAfriVac catch-up campaigns of 1–6-year-olds (2016) and routine immunisation of 15–18-month-olds (starting in 2017).^11^

**Research in context**

**Evidence before this study**

We searched PubMed with the terms ([meningococcal OR *Neisseria meningitidis*] AND carriage AND meningococcal vaccines AND Africa), with no date or language restrictions, on Sept 25, 2019, to identify meningococcal carriage surveys done in the meningitis belt of sub-Saharan Africa. Although the impact of a serogroup A meningococcal conjugate vaccine (MenAfriVac) on serogroup A meningococcal carriage has been described up to 2 years after vaccine introduction in Burkina Faso, resulting in near-elimination of disease and induction of herd immunity, the long-term effect of the vaccine on serogroup A meningococcal carriage remains unknown.

**Added value of this study**

In our evaluation, 7 years since MenAfriVac was first introduced in Burkina Faso through a mass vaccination campaign of 1–29-year-olds, no serogroup A meningococcal carriage was identified. These findings show, to our knowledge for the first time, the long-term effect of MenAfriVac on serogroup A carriage. Despite large-scale epidemics of serogroup C meningococcal meningitis in neighbouring countries, the prevalence of epidemic-prone *N meningitidis* strains was low in our study. Additionally, cross-sectional studies done over an 8-year period in Kaya district offer a unique insight into the dynamic nature of meningococcal carriage in the meningitis belt, with evidence of clonal carriage waves, most recently due to capsule-null non-groupable sequence type 192 strains.

**Implications of all the available evidence**

Results from this evaluation as well as surveillance data show that the strategy of vaccinating a large proportion of the population with MenAfriVac through a mass vaccination campaign was effective in reducing carriage and disease, as well as inducing herd immunity in unvaccinated cohorts for at least 7 years after vaccine introduction. These findings will be useful to help inform the vaccination strategy for and measure the effect of future multivalent meningococcal vaccines currently under development for use in Africa.

## Methods

### Study design

**Figure 1:**
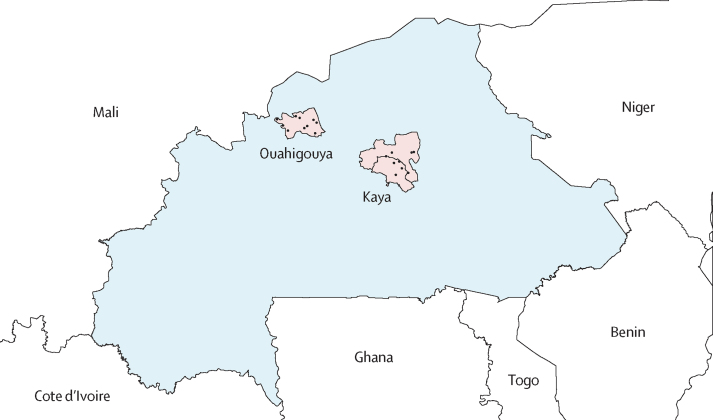
Map of meningococcal carriage evaluation sites Each black dot corresponds to a village where carriage evaluation was done. In 2016, the district of Kaya split into two districts to form the new district of Boussouma; however, for the purposes of this evaluation, Kaya district boundaries correspond to the original Kaya district before subdivision of the district into Kaya and Boussouma districts.

We did four cross-sectional carriage surveys in the rural districts of Kaya and Ouahigouya in Burkina Faso between May 2, 2016, and Nov 6, 2017: two per district in the dry (April–May) and rainy (October–November) seasons (figure 1). During each survey round, all data were collected over a 4-week period. Study procedures were harmonised with those from a previous carriage evaluation in Burkina Faso from 2009 to 2012, which included Kaya district.^12^ In Ouahigouya, ten villages (eight target and two reserve) were selected by probability proportional to size sampling; in Kaya, the ten villages selected by probability proportional to size sampling in the 2009–12 evaluation were retained (appendix p 2). Within each village, study personnel enumerated and geocoded all compounds, defined as a group of people who had the same head of household and prepared meals in a single area. 40 compounds per village were selected through simple random sampling before each survey round, and compounds were recruited until a sample size of approximately 210 participants from each of the eight target villages per district was reached, resulting in an estimated total sample size of 3360 participants (1680 per district, consistent with the previous evaluation^12^) per survey round, or 13 440 participants in total.

This study was approved by the Ethical Committee for Health Research in Burkina Faso and was determined by human subjects review at the Centers for Disease Control and Prevention (CDC) to be public health non-research; therefore, CDC and Norwegian Institute of Public Health (NIPH) institutional review board review was not required. Voluntary participation was explained to all participants or their guardian in local languages by trained study staff. Written informed consent was obtained from participants, or in the case of children younger than 18 years from the parent or guardian, through either signature or thumbprint.

### Data and specimen collection

Community sensitisation activities were done before the start of the evaluation in each selected village. During each survey round, study personnel visited selected compounds to provide information and recruit eligible participants. All people aged 9 months to 36 years (the youngest age recommended by WHO for routine MenAfriVac immunisation^13^ and the oldest age eligible for the 2010 MenAfriVac mass vaccination campaign at the start of the evaluation) from selected compounds were invited to participate.

Household demographic information was collected from the head of household and individual demographic, behaviour, and clinical information was collected for each participant using CommCare, a mobile data collection platform.^14^ Consenting participants were given a paper wristband with a barcoded unique identifier and were referred to a centralised specimen collection site in the village.

Oropharyngeal swabs from the posterior pharynx and one tonsil were collected by trained personnel using a sterile cotton swab (Copan Italia, Brescia, Italy). Swabs were immediately inoculated and streaked on modified Thayer-Martin media containing vancomycin, colistin, nystatin, trimethoprim, and Vitox supplement (produced at the WHO Intercountry Support Team Laboratory, Ouagadougou, Burkina Faso), assigned a barcoded unique identifier, and stored in CO_2_-rich Mitsubishi AnaeroPack rectangular jars (Thermo Fisher Scientific, Waltham, MA, USA) at ambient temperature, maintained at less than 37°C, along with a negative control plate. The bar codes on the patients' wristbands and inoculated plates were scanned to link participant and specimen data, and data were uploaded into a study database automatically through cloud-based synchronising. Inoculated plates were transported to the regional hospital laboratories in Kaya and Ouahigouya as soon as possible after collection, with a maximum delay of 6 h.

### Laboratory methods

At the regional laboratories, plates were incubated at 37°C (5% CO_2_) and examined for bacterial growth at 24 h and 48 h. Colonies with neisserial morphology were subcultured on blood agar (Thermo Fisher Scientific, Waltham, MA, USA and Centre Hospitalier Universitaire de Bogodogo, Ouagadougou, Burkina Faso). *N meningitidis* was identified using Gram stain, oxidase test (BD, Franklin Lakes, NJ, USA), o-nitrophenyl-β-D-galactoside test (ONPG; Rosco Diagnostica, Taastrup, Denmark), and gamma-glutamyl transferase test (GGT; Rosco Diagnostica). Presumptive meningococcal isolates (eg, oxidase positive, Gram-negative diplococci, ONPG negative, and GGT positive) were tested by slide agglutination serogrouping to measure the expression of the capsular polysaccharide for serogroups A, C, W, X, Y, and Z (Thermo Fisher Scientific, Remel Products, Lenexa, KS, USA). An isolate was defined as non-groupable by slide agglutination when it autoagglutinated, polyagglutinated, or did not agglutinate. Purified isolates were stored in cryovials containing 0·5 mL of Greaves solution (produced at the NIPH, Oslo, Norway) at –80°C.^15^ Epidemiological and laboratory data were linked via the barcoded unique identifiers in the study database.

Internal quality-control procedures were done on media, reagents, laboratory equipment, and field conditions, as previously described.^16^ External quality control was done on a subset of collected specimens at NIPH to assure the sensitivity and specificity of *N meningitidis* identification using the same methods used during field testing. These included 50 plates per district per round with non-neisserial morphology, all isolates that were oxidase positive with Gram-negative diplococci and were ONPG positive and GGT positive, all isolates that were ONPG negative and GGT negative, and 10% of presumptive *Neisseria lactamica* isolates (ONPG positive and GGT negative).

Additional confirmation and characterisation of presumed meningococcal isolates were done at NIPH and the CDC Bacterial Meningitis Laboratory using whole-genome sequencing (WGS). At NIPH, isolates were tested using oxidase, ONPG, GGT, and matrix-assisted laser desorption/ionisation–time of flight. For WGS, DNA was extracted using a MagNA Pure isolation station and MagNA Pure 96 DNA and Viral NA Small Volume Kit (Roche, Basel, Switzerland), according to the manufacturer's instructions. Sequencing libraries were prepared using KAPA HyperPlus Kit (KAPA Biosystems, Wilmington, MA, USA) with size selection of fragments 450 bp or smaller. At the CDC Bacterial Meningitis Laboratory, isolates were tested for species by real-time PCR for *sodC* and the API NH strip system (bioMérieux, Marcy L'Étoile, France),^17^ with WGS done using Prepito (PerkinElmer, Waltham, MA, USA). Illumina sequencing libraries were prepared by shearing extracted DNA using Covaris LE220 focused ultrasonicator (Covaris, Woburn, MA, USA) to 600 bp. Sheared DNA was subsequently processed using the NEBNext ultra DNA library preparation kit (New England Biolabs, Ipswich, MA, USA) in accordance with the manufacturer's protocol.

Genomic DNA samples were sequenced on either Illumina Miseq or HiSeq 2500 using 250 bp paired-end sequencing (Illumina, San Diego, CA, USA). The sequence read files were trimmed with cutadapt to remove adaptor sequences and any reads with a sequence quality below 28.^18^ Assemblies were produced using the de-novo assembly software SPAdes 3.7.0.^19^ The genome assemblies were used to characterise sequence type (ST) clonal complex (CC) and PorA and FetA types of the isolate by comparing individual genes or peptide sequences against PubMLST.^20^ The serogroup of each isolate was defined using WGS-based methods, which consisted of characterising the capsule locus for each isolate and investigating each identified capsule gene for genetic variations (eg, mutations, gene deletions, or insertions) to predict the capsule phenotype, as described previously.^21^ Sequences from isolates analysed at NIPH were sent to the CDC for parallel analysis. WGS-based capsule results were compared against the country's slide agglutination serogrouping results for confirmation; in case of discrepancy between laboratories, the isolate underwent retesting by slide agglutination serogrouping at NIPH or CDC until agreement was reached.

### Statistical analysis

Prevalence and 95% CIs of meningococcal carriage in Kaya and Ouahigouya during 2016–17 were estimated overall and by district, season, age, sex, and serogroup. Additionally, meningococcal carriage prevalence and serogroup distribution were assessed in Kaya from 2009 to 2017 by analysis of data collected in previous carriage rounds using harmonised epidemiological and laboratory methods done by the same study partners.^3^, ^4^, ^12^ To account for the complex survey design, all analyses were done using the survey analysis procedures in SAS (version 9.4), with districts as survey strata, villages as primary sampling units (clusters), and sampling weights calculated as the reciprocal of each sample member's probability of selection into the sample. The weight calculations assume that all eligible people in each compound were surveyed.

### Role of the funding source

The sponsors of the study had no role in study design, data collection, data analysis, data interpretation, or writing of the report. The corresponding author had full access to all the data in the study and had final responsibility for the decision to submit for publication.

## Results

Of 14 295 eligible people invited to participate, 14 290 (>99·9%) consented; of these, specimens were collected from and laboratory results were available for 13 762 (96·3%). Isolates from four participants with a preliminary result of *N meningitidis* from field testing in Burkina Faso were excluded from the analysis because no isolate was available for confirmatory testing at the CDC or NIPH. Thus, 13 758 participants with specimens were included in the analysis (appendix p 3). Results of external quality-control testing showed high performance of the laboratory testing in Burkina Faso (appendix p 4).

**Figure 2:**
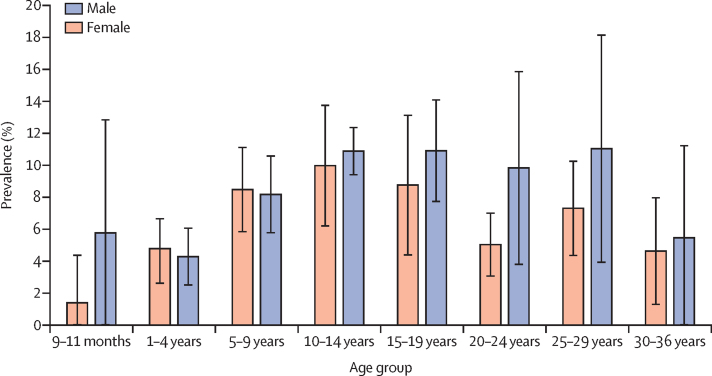
Meningococcal carriage prevalence by age and sex Bars are 95% CIs. Carriage prevalence estimates account for complex survey design.

1035 meningococcal carriage isolates were recovered and confirmed, for a carriage prevalence of 7·60% (95% CI 5·67–9·52; table). Carriage was highest in sampling round 3 and lowest in round 1. By district, carriage prevalence was 5·44% (95% CI 4·18–6·69) in Ouahigouya and 9·14% (6·01–12·27) in Kaya. By age group, carriage prevalence ranged from 3·39% (95% CI 0·00–8·30) in infants aged 9–11 months to 10·43% (8·08–12·79) in adolescents aged 10–14 years (table, figure 2). Carriage prevalence was similar by season and sex (table). Non-groupable meningococci accounted for 90·3% of all carriage, with a prevalence of 6·98% (95% CI 4·86–9·11). Prevalence of encapsulated meningococci was low: 0·48% (95% CI 0·01–0·95) for serogroup W, 0·10% (0·01–0·18) for serogroup C, and 0·03% (0·00–0·80) for serogroup E (table). No carriage due to serogroups A, B, X, Y, or Z was detected.

**Table:** Participant characteristics and prevalence of meningococcal carriage

		**Participants**	Carriage prevalence (95% CI)*
Overall		13 758 (100%)	7·60% (5·67–9·52)
District			
	Kaya	6833 (49·7%)	9·14% (6·01–12·27)
	Ouahigouya	6925 (50·3%)	5·44% (4·18–6·69)
Round			
	1	3415 (24·8%)	4·67% (2·71–6·64)
	2	3467 (25·2%)	4·69% (3·39–5·99)
	3	3467 (25·2%)	11·17% (6·75–15·59)
	4	3409 (24·8%)	9·72% (4·77–14·66)
Season			
	Dry	6882 (50·0%)	8·03% (6·27–9·79)
	Rainy	6876 (50·0%)	7·20% (4·78–9·62)
Age group			..
	9–11 months	189 (1·4%)	3·39% (0·00–8·30)
	1–4 years	3536 (25·7%)	4·53% (2·61–6·45)
	5–9 years	4439 (32·3%)	8·33% (5·86–10·80)
	10–14 years	2824 (20·5%)	10·43% (8·08–12·79)
	15–19 years	1122 (8·2%)	9·56% (6·07–13·06)
	20–24 years	619 (4·5%)	6·08% (3·86–8·30)
	25–29 years	539 (3·9%)	8·08% (5·19–10·98)
	30–36 years	490 (3·6%)	4·86% (1·53–8·19)
Sex			
	Female	7267 (52·8%)	7·40% (5·11–9·69)
	Male	6491 (47·2%)	7·82% (6·21–9·43)
Serogroup			
	C	13 758 (100%)	0·10% (0·01–0·18)
	E	13 758 (100%)	0·03% (0·00–0·80)
	W	13 758 (100%)	0·48% (0·01–0·95)
	Non-groupable	13 758 (100%)	6·98% (4·86–9·11)

* Estimates account for complex survey design.

**Figure 3:**
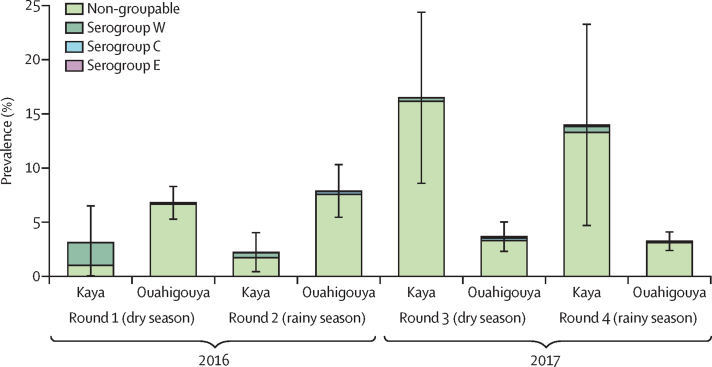
Meningococcal prevalence by sampling round, district, and serogroup Bars are 95% CIs. Carriage prevalence estimates account for complex survey design.

Differences in meningococcal carriage prevalence and serogroup distribution were observed by district and sampling round (figure 3, appendix p 5). In 2016, carriage prevalence in Kaya was low (3·12% [95% CI 0·00–6·56] in round 1 and 2·23% [0·38–4·09] in round 2), whereas it was higher in 2017 (16·48% [8·54–24·43] in round 3 and 13·98% [4·64–23·32] in round 4). In round 1 in Kaya, serogroup W predominated, with a prevalence of 2·09%, decreasing to 0·33–0·55% in rounds 2–4 (figure 3). During these later rounds, most meningococcal carriage isolates were non-groupable, with a prevalence of 1·72–16·15%. At the village level in Kaya, overall *N meningitidis* carriage prevalence ranged from 4·82% to 15·14%; however, most serogroup W isolates were recovered from two villages (81·7%). In Ouahigouya, carriage prevalence was 6·78% (95% CI 5·22–8·35) in round 1 and 7·88% (5·40–10·37) in round 2 in 2016, and declined to 3·67% (2·26–5·07) in round 3 and 3·24% (2·33–4·16) in round 4 in 2017. In all rounds in this district, most meningococcal carriage isolates were non-groupable, with a prevalence of 3·11–7·57% (figure 3). At the village level in Ouahigouya, overall carriage prevalence ranged from 2·96% to 9·96%; 55·6% of serogroup C isolates were recovered from the same village.

Among 1035 meningococcal carriage isolates, 21 sequence types from seven clonal complexes, including new or unassigned sequence types or clonal complexes, were identified (appendix p 6). All 83 serogroup W isolates belonged to CC11. Nine (69%) of 13 serogroup C isolates were CC10217 and four (31%) were CC41/44. Among 934 non-groupable isolates, 822 (88%) were CC192, 89 (10%) were CC175, 13 (1%) were other, and ten (1%) were unassigned. 832 (89%) non-groupable isolates (80% of all meningococcal isolates) were capsule null. No serogroup A-associated sequence types were identified among isolates of any serogroup. Further characterisation of meningococcal isolates for sequence type and PorA and FetA types are shown in the appendix (p 6).

**Figure 4:**
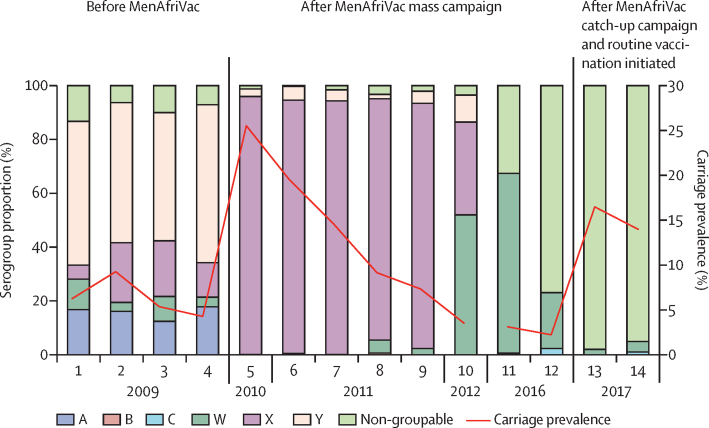
Meningococcal carriage prevalence and serogroup distribution by sampling round Carriage prevalence estimates account for complex survey design.

Among 23 525 specimens collected from participants in Kaya district from 2009 to 2017, overall carriage prevalence ranged from 2·2% to 25·5% by sampling round (figure 4). During the four sampling rounds in 2009—before MenAfriVac mass vaccination in Kaya—serogroup A carriage prevalence ranged from 0·67% to 1·49% (0·93% overall during the pre-vaccination period) and accounted for 12·4–16·8% of all meningococcal carriage; serogroup Y was the predominant serogroup, accounting for 47·7–58·7% of carriage isolates during this time (figure 4). In the ten post-vaccination sampling rounds from 2010–12 and 2016–17, no serogroup A carriage was detected. In 2010–11, serogroup X predominated, accounting for 89·6–96·0% of carriage. In 2012, serogroup W accounted for 52% of carriage and remained the predominant serogroup during round 1 of 2016 at 66·9%. By round 2 of 2016 and into 2017, non-groupable *N meningitidis* was the dominant serogroup, at 77·0–98·0%.

## Discussion

7 years after MenAfriVac was first introduced in Burkina Faso through mass vaccination of 1–29-year-olds, no serogroup A meningococcal carriage was identified, even among unvaccinated cohorts up to 6 years of age before implementation of catch-up campaigns and routine immunisation. These findings suggest MenAfriVac has long-term effects on carriage of serogroup A *N meningitidis*, with carriage declining from an average of approximately 1% of the population pre-vaccination to 0% of the population in the 7 years after the mass vaccination campaign in Kaya district. Over 90% of meningococcal isolates from 2016 to 2017 were non-groupable, predominantly capsule null, and the prevalence of epidemic-prone strains, such as serogroup W ST-11 and serogroup C ST-10217, was low.

Consistent with other evaluations in the African meningitis belt,^22^, ^23^ carriage varied by age, with the highest prevalence among adolescents and numerically higher prevalence in male compared with female young adults. By contrast with previous evaluations in Burkina Faso,^4^, ^12^ carriage prevalence did not appear to be higher in the dry season than in the rainy season. Although the reason for this finding is unknown, it might reflect differences in transmission dynamics between encapsulated and unencapsulated carriage strains in the meningitis belt. Additionally, apparent differences in carriage prevalence and serogroup distribution by district probably reflect the dynamic nature of meningococcal carriage, which fluctuates over time and varies geographically owing to patterns of local transmission.

Consistent with the absence of detection of serogroup A carriage among the nearly 14 000 oropharyngeal specimens collected in our evaluation, only six cases of invasive serogroup A meningococcal meningitis, including one in a vaccinated individual, were reported through Burkina Faso's nationwide case-based meningitis surveillance system from the end of the mass vaccination campaign in 2010 to 2018; all were reported before MenAfriVac was introduced in the routine immunisation programme.^6^, ^24^, ^25^ Furthermore, a serological evaluation in Burkina Faso using rabbit complement serum bactericidal activity titres^26^ showed persistence of protective anti-serogroup A antibodies for at least 5 years after the mass vaccination campaign, with predicted persistence for 8–12 years depending on age at vaccination. Taken together with the findings from our study, these findings suggest that MenAfriVac is effective in protecting against invasive meningococcal disease and carriage and promoting herd immunity for at least 7 years after completion of the vaccination campaign. However, mathematical models predict a return of serogroup A disease and epidemics if further vaccination activities are not implemented within 15 years of mass vaccination, underscoring the need for implementation of routine MenAfriVac immunisation with high coverage to sustain the gains afforded by MenAfriVac so far.^27^, ^28^

The predominance of capsule-null non-groupable *N meningitidis* in our study is in stark contrast with results from previous carriage evaluations in Burkina Faso from 2009 to 2012, in which non-groupable strains accounted for only 1·5–16·5% of isolates by survey round.^3^, ^12^, ^29^ Because the proportion of strains that were non-groupable remained reasonably consistent in the 2 years before and after MenAfriVac mass vaccination, and the increase in non-groupable carriage occurred before MenAfriVac catch-up campaigns and routine immunisation, this finding is unlikely to be related to MenAfriVac implementation. Carriage studies done in seven other meningitis belt countries showed that over half of isolates were unencapsulated, but the proportion varied widely by country and was similarly unaffected by MenAfriVac implementation.^23^ Nearly all non-groupable isolates in our evaluation, done over a 2-year period in two non-contiguous districts of Burkina Faso, were ST-192, with little variation in PorA type, suggesting a clonal wave. However, with only 2 years of data, we cannot establish whether this finding is transient or indicates stable replacement of encapsulated organisms with unencapsulated ones. Carriage of ST-192 or the genogroup and PorA type combination most commonly associated with ST-192 has been observed in several other African countries in recent years.^23^, ^30^ Further evaluation is needed to establish whether this finding persists, which has implications for the epidemiology of invasive meningococcal meningitis and future vaccination strategies.

A pattern of apparent clonal waves of meningococcal carriage was observed in Kaya from 2009 to 2017,^29^ in which the predominance of serogroup Y ST-23 gave way to serogroup X ST-181, followed by serogroup W ST-11, and then non-groupable ST-192. Similar clonal waves were observed in a longitudinal study in Ghana,^31^ and the investigators postulated that the absence of a stable and genetically diverse pharyngeal flora contributes to rapid propagation of novel clones in an immunologically naive population (ie, without previous exposure to these clones and thus without population-level immunity). This hypothesis seems to be supported by the large-scale serogroup C epidemics in Niger and Nigeria that were caused by a novel ST-10217 strain; little to no serogroup C carriage was detected in Niger and Nigeria in studies done 3–5 years before the outbreak during 2010–12, and serological evaluations in Niger showed that population immunity to serogroup C was low.^23^, ^32^ The causative strain in these outbreaks probably evolved from a non-groupable strain, isolated in 2012 during the previous carriage evaluation in Burkina Faso, that acquired capsule genes and virulence factors, highlighting the contribution of carriage studies to molecular surveillance in the meningitis belt.^33^

The predominance of carriage of non-groupable meningococcal strains, which rarely cause invasive disease in healthy people,^34^ followed by carriage of serogroup W strains is consistent with the epidemiology of invasive meningococcal meningitis observed in Burkina Faso during the evaluation period: the incidence of laboratory-confirmed meningitis (1·3 cases per 100 000 population) was low compared with the previous 5 years in Burkina Faso and with several other meningitis belt countries, with serogroup W the leading cause of invasive disease.^6^, ^24^ However, the epidemiology of meningococcal meningitis in this region is dynamic and evolving. Large-scale epidemics of serogroup C and W meningococcal meningitis, along with increases in cases due to serogroup X, have been reported in neighbouring countries.^8^, ^9^, ^24^ New meningococcal conjugate vaccines targeting multiple serogroups are under development, with potential for future use in sub-Saharan Africa.^35^ Additional carriage evaluations and high-quality meningitis surveillance will be crucial to help inform vaccination policy in Burkina Faso and elsewhere in the meningitis belt.

This study was limited by the substantial logistical challenges necessary to obtain bacterial culture from a large number of specimens in remote, resource-limited settings, thereby precluding recruitment of a larger sample size in additional geographical locations. Development of novel, non-culture-based methods that permit detailed characterisation of meningococcal carriage would help improve the efficiency and scalability of future carriage studies to assess the effect of the next generation of meningococcal vaccines in Africa. Additionally, the cross-sectional nature of our study did not permit a detailed evaluation of household or community transmission patterns, which might help inform additional strategies to halt transmission in high-burden settings. Finally, the differences in carriage prevalence and serogroup distribution by district suggest that the results are not necessarily representative of Burkina Faso as a whole or other countries of the meningitis belt.

Results from our carriage evaluation in Burkina Faso show the long-term effect of MenAfriVac on serogroup A carriage, with an absence of serogroup A carriage identified 7 years after the mass MenAfriVac campaign, as well as the dynamic nature of meningococcal clonal waves in the meningitis belt. Although these findings, along with surveillance data, affirm the effectiveness of MenAfriVac and the vaccination strategy, further evaluations and modelling of potential vaccination strategies are needed to inform vaccination policy for future multivalent meningococcal vaccines, as well as to evaluate the effect of these vaccines on disease, carriage, and herd immunity.
