# Supplementary material for: Meningococcal carriage 7 years after introduction of a serogroup A meningococcal conjugate vaccine in Burkina Faso: results from four cross-sectional carriage surveys
Source: Lancet Infect Dis. 2020 Dec;20(12):1418–25. doi: 10.1016/S1473-3099(20)30239-5 (PMC7689286; doi:10.1016/S1473-3099(20)30239-5)
Supplement: Supplementary appendix [file mmc1.pdf]

THE LANCET
Infectious Diseases

Supplementary appendix

This appendix formed part of the original submission and has been peer reviewed. We post it as supplied by the authors.

Supplement to: Mbaeyi S, SampoE, DinanibK, et al. Meningococcal carriage 7 years after introduction of a serogroup A meningococcal conjugate vaccine in Burkina Faso: results from four cross-sectional carriage surveys. *Lancet Infect Dis* 2020; published online July 9. https://doi.org/10.1016/S1473-3099(20)30239-5.

Meningococcal carriage 7 years after introduction of a serogroup A meningococcal conjugate vaccine in Burkina Faso: results from four cross-sectional carriage surveys

Appendix

Contents

Supplemental Figure 1. Detailed maps of Kaya and Ouahigouya villages and carriage evaluation sites – Burkina Faso, 2016–2017. 2

Supplemental Figure 2. Schematic of carriage evaluation participant recruitment – Burkina Faso, 2016–2017.. 3

Supplemental Table 1. Results of external quality control (EQC) testing by round – Burkina Faso, 2016–2017. 4

Supplemental Table 2. Prevalence of meningococcal carriage by district – Burkina Faso, 2016–2017.. 5

Supplemental table 3. Molecular typing profile of meningococcal isolates – Kaya and Ouahigouya districts, 2016–2017. 6

Supplemental Figure 1. Detailed maps of Kaya and Ouahigouya villages and carriage evaluation sites – Burkina Faso, 2016–2017.

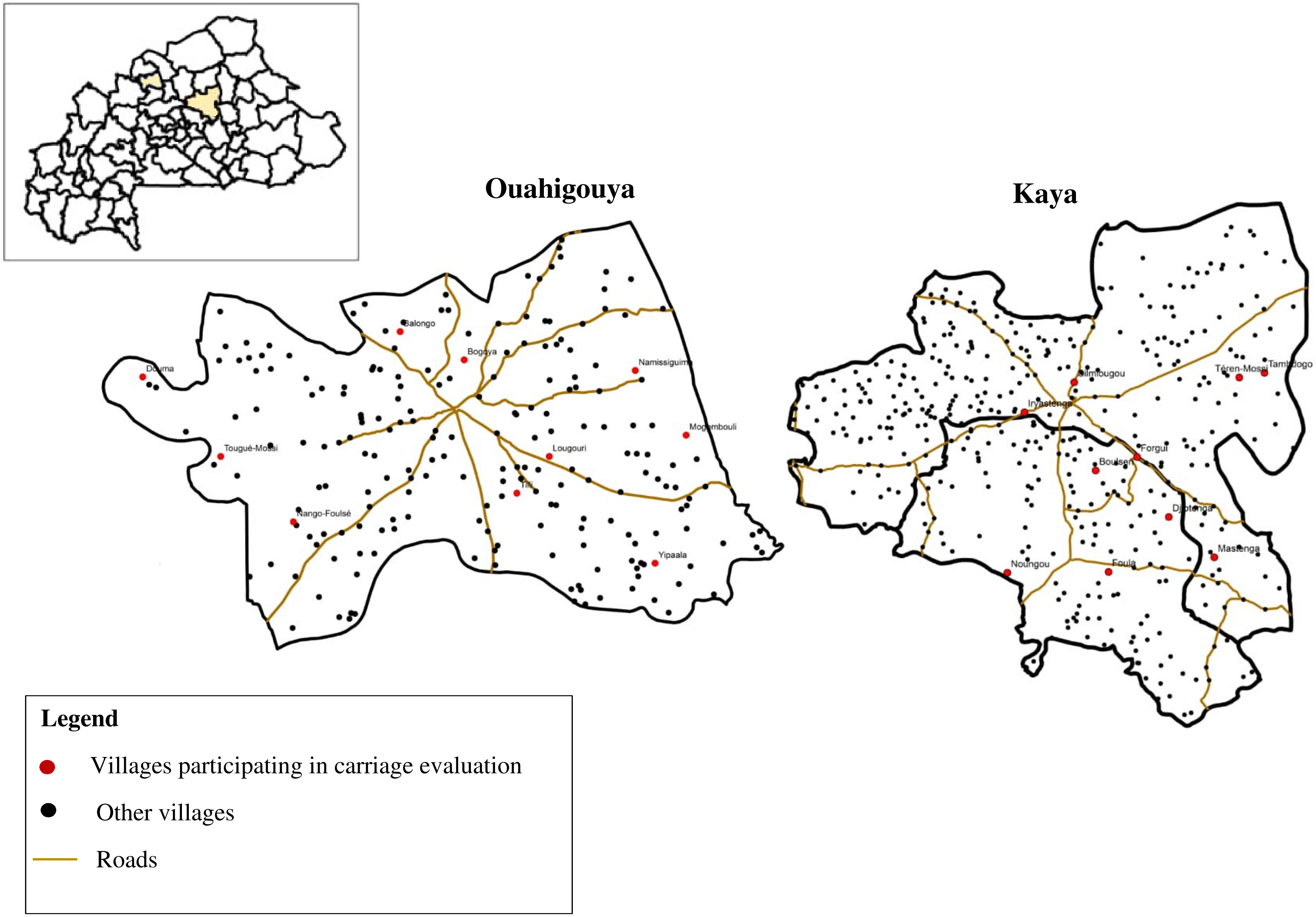

In 2016, the district of Kaya split into two districts to form the new district of Boussouma; however, for the purposes of this evaluation, Kaya district boundaries corresponds to the original Kaya district before subdivision of district into Kaya and Boussouma districts.

Supplemental Figure 2. Schematic of carriage evaluation participant recruitment – Burkina Faso, 2016–2017.

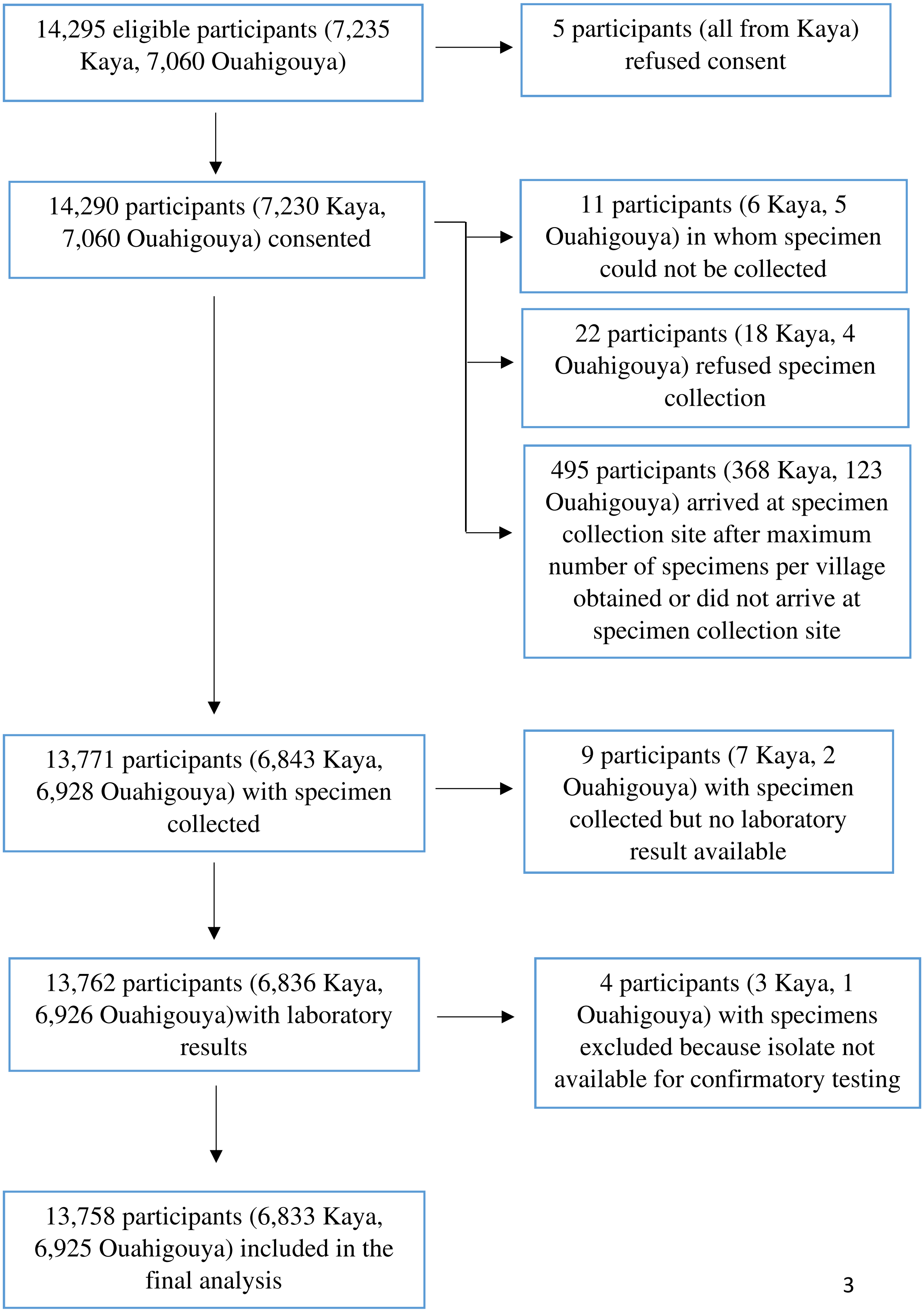

Supplemental Table 1. Results of external quality control (EQC) testing by round – Burkina Faso, 2016–2017.

	Round 1	Round 2	Round 3	Round 4
N. samples analyzed	3,415	3,467	3,467	3,409
**Morphology step: screening of samples where no colonies morphologically resembling *N. meningitidis* identified**				
N. samples with suspect colonies[a]	1,332	986	979	1,007
N. samples without suspect colonies	2,083	2,481	2,488	2,402
N. samples without suspect colonies analyzed by EQC	50	50	50	50
N. *N. meningitidis*	0	0	0	0
**Galactosidase (ONPG)/Glutamyl transferase (GGT) activity step: screening of isolates with enzymatic profile different from Neisseria meningitidis (ONPG−/GGT+)**				
N. oxidase-positive, Gram-negative diplococci analyzed	771	860	847	873
N. ONPG−/GGT+ isolates	242	211	352	327
N. ONPG+/GGT+ or ONPG−/GGT− isolates	105	205	139	64
N. ONPG+/GGT+ or ONPG−/GGT− isolates analyzed by EQC	105	205	139	64
N. *N. meningitidis*	13	0	0	0
**Sensitivity and specificity of laboratory analysis performed in Burkina Faso**				
Sensitivity [b]	93.0%	99.4%	99.7%	99.4%
Specificity [c]	97.8%	98.9%	99.9%	99.8%

[a] Suspect colonies are colonies growing on selective agar morphologically resembling *N. meningitidis*.

Sensitivity and specificity for *N. meningitidis* identification calculated by comparing preliminary results from laboratory testing in Burkina Faso to the final results from EQC testing:

Sensitivity= number of true positives/(true positives+false negatives)

Specificity=number of true negatives/(true negatives+false positives)

Supplemental Table 2. Prevalence of meningococcal carriage by district – Burkina Faso, 2016–2017.

Characteristic	Kaya		Ouahigouya	
	Carriage prevalence* (%)	95% CI	Carriage prevalence* (%)	95% CI
**Overall**	9·14	6·01–12·27	5·44	4·18–6·69
**Round**				
1	3·12	0·00–6·56	6·78	5·22–8·35
2	2·23	0·38–4·09	7·88	5·40–10·37
3	16·48	8·54–24·43	3·67	2·26–5·07
4	13·98	4·64–23·32	3·24	2·33–4·16
**Season**				
Dry	10·08	6·88–13·29	5·19	3·84–6·54
Rainy	8·28	3·77–12·80	5·67	3·80–7·54
**Age group**				
9–11 months	5·19	0·00–16·26	1·78	0·00–4·43
1–4 years	5·90	2·33–9·46	2·48	1·51–3·44
5–9 years	10·11	5·87–14·35	5·14	2·99–7·28
10–14 years	11·20	7·54–14·86	9·30	5·70–12·89
15–19 years	10·89	3·39–18·40	8·21	6·14–10·27
20–24 years	7·08	2·41–11·74	5·12	3·24–6·99
25–29 years	11·83	5·39–18·26	5·04	1·51–8·58
30–36 years	8·75	0·43–17·07	3·28	0·09–6·47
**Sex**				
Female	8·89	4·66–13·12	5·36	3·30–7·42
Male	9·42	6·42–12·42	5·53	4·31–6·75
**Serogroup**				
C	0·06	0·00–0·11	0·15	0·00–0·35
E	0·00	..	0·08	0·00–0·20
W	0·82	0·00–1·71	0·01	0·00–0·02
Nongroupable	8·26	4·28–12·24	5·20	3·75–6·65

*Carriage prevalence estimates accounting for complex survey design

Supplemental table 3. Molecular typing profile of meningococcal isolates – Kaya and Ouahigouya districts, 2016–2017.

Serogroup	Clonal complex	Sequence type	PorA	FetA	N. isolates
C	CC10217	10217	P1.21-15;16	F1-7	4
		12446			3
		13402			2
	CC41/44	7376	P1.18-3;1	F1-100	4
E	CC178	188	P1.19;15	F1-18	2
		13407			1
	Unassigned	9945	P1.21;16-14	F3-6	2
W	CC11/ET-37	11	P1.5;2	F1-1	81
			P1.5;2-67		2
NG	CC192	192	P1.18-11;42-1	Not present	647
			P1.18-46;42-1		61
			P1.18-11;42-11		18
			New		3
			P1.18-11;42-8		2
			P1.18-11;42-2		1
			P1.18-11;42-3		1
			P1.18-11;42-5		1
			P1.Peptide not found;42		1
		13199	P1.18-11;42-1		77
		13404			7
		14388			1
		14389			1
		13802	New		1
	CC175	175	P1.22-11;15-25	F5-1	84
				F1-31	1
		2881	P1.5-1;2-36	F5-1	2
			P1.5-1;2-74		2
	CC11/ET-37	11	P1.5;2	F1-1	6
	CC178	13407	P1.19;15	F1-18	2
		188			1
	CC10217	10217	P1.21-15;16	F1-7	1
		13402			1
	CC35	35	P1.22-1;14	F4-1	1
	CC41/44	7376	P1.18-3;1	F1-100	1
	Unassigned	6924	P1.21-14;28-3	F5-66	5
		12718			1
		8248			1
		13386	P1.22;14-6		1
		9945	P1.21;16-14	F3-6	1
		13402	P1.22;14-6	F5-66	1
